# Editorial: Bioengineered nanoparticles in cancer therapy, Volume III

**DOI:** 10.3389/fmolb.2024.1356081

**Published:** 2024-02-22

**Authors:** Tomy J. Gutiérrez

**Affiliations:** Grupo de Nanotecnología de Alimentos y Agro-Alimentos (NanoÅ^2^), Departamento de Química, Facultad de Ciencias Exactas y Naturales, Universidad de Belgrano (UB) y Consejo Nacional de Investigaciones Científicas y Técnicas (CONICET), Capital Autónoma de Buenos Aires (CABA), Argentina

**Keywords:** bioengineering, biomaterials, cancer therapy, nanoparticles, nanothechnology

Nanotechnology has brought great advances in different fields, and medicine has not escaped from this (Zarrintaj et al., 2019). In this regard, the development of different treatments and devices for the treatment and timely detection of cancer today passes through this area (Zarrintaj et al., 2022). Cancer is a wide term, which is related to the quick growth of abnormal cells in organs or tissues. According to the World Health Organization (WHO, 2023), 20% of the world’s population will suffer from some type of cancer throughout their lives. However, the incidence of cancer can be reduced between 30% and 50% by reducing risk factors and applying preventive strategies based on scientific evidence (WHO, 2023). Nonetheless, this does not make said disease disappear, which is the second cause of death worldwide (WHO, 2023). With this in view, our goal with this Research Topic was to continue highlighting research in the field of anticancer therapies based on nanoparticles (NPs). Then, the use of four different NP systems based on carbon, graphene oxide, selenium and silver for the treatment of diverse types of cancer such as carcinoma, osteosarcoma, rectal cancer and breast cancer can be highlighted in this issue. For instance, Lu et al. synthesized ginsenoside Rg3-loaded graphene oxide NPs as a non-invasive photodynamic therapy for the treatment of osteosarcoma. These authors found that osteosarcoma cells were inhibited *in vivo* by the aforementioned NPs, thus improving their cell apoptosis. Meanwhile, Abo-Neima et al. synthesized selenium NPs for their evaluation alone or assisted by radiotherapy (2 mW laser power) against human colon cancer cell line (HCT-116) and Ehrlich ascites carcinoma. As relevant results of this research, maximum inhibition values of cancer cells tested *in vitro* were found between 73% and 80.57% and IC50 = 14.86 and 50 mg/mL. In addition to this, Abo-Neima et al. also evidenced that selenium NPs were non-toxic, and had high efficacy as a photothermal agent in radiotherapy, resulting in a reduction in tumor volume and massive necrosis of tumor cells. On the other hand, Pi et al. found that carbon NPs can indirectly contribute to reducing colon cancer recurrence rates by reducing the lymph node ratio and significantly increasing the use of total lymph nodes. However, the authors highlight that more studies must be conducted to elucidate the role of carbon NPs on colorectal cancer. For now, this study showed that carbon NPs can help in predicting recurrence of stage III rectal cancer. Lastly, Maher et al. evaluated the effect of silver NPs coated by an alginate-based hydrogel containing cisplatin on synergistic cytotoxicity in breast cancer cells. These composite NPs showed high efficacy against breast cancer cells compared to silver NPs or cisplatin as stand-alone treatments, i.e., synergistic action of the composite NPs could potentially reduce the side effects associated with high doses of cisplatin as a stand-alone treatment. This editorial closes by once again highlighting the importance of NPs in cancer therapies and illuminating an even more complex field in cancer treatment such as bionanosystems. Bionanosystems in the near future will aid much more in anticancer treatments than the simple development of anticancer NPs, since these would be designed with finer chemistry to emulate living systems, even assisted with artificial intelligence.
